# Selective Peripheral Denervation and Selective Nerve Injury for the Treatment of Cervical Dystonia Through a Periauricular Incision

**DOI:** 10.7759/cureus.58239

**Published:** 2024-04-14

**Authors:** Brian Mailey, Blake M Sparkman, Alina K Sinha, Timothy Daugherty, Kevin Calder

**Affiliations:** 1 Institute for Plastic Surgery, Southern Illinois University School of Medicine, Springfield, USA; 2 Plastic and Reconstructive Surgery, Saint Louis University School of Medicine, Saint Louis, USA; 3 Plastic and Reconstructive Surgery, University of Missouri–Kansas City School of Medicine, Kansas City, USA; 4 Plastic and Reconstructive Surgery, University of North Carolina at Chapel Hill School of Medicine, Chapel Hill, USA

**Keywords:** sunderland nerve injury, periauricular incision, peripheral denervation, torticollis, retrocollis, cervical dystonia

## Abstract

Traditional selective peripheral denervation methods for treating cervical dystonia (CD) involve complete transection of the nerves to muscles through a posterior incision proximally after they exit the spinal cord. This report presents a case where anterior muscles involved in CD cannot be easily addressed through the traditional posterior approach. Furthermore, complete denervation of certain muscles, such as the trapezius, can lead to functional limitations. The objective of this report is to describe an anterior surgical treatment approach for focal CD. Specifically, we describe the use of a periauricular incision to perform selective peripheral denervation of anterior and posterior neck muscles at a more peripheral location near their target muscle entry point. Complete denervation was performed for expendable muscles while Sunderland third-degree nerve injury was performed to weaken nonexpendable muscles. This approach facilitates clearer identification of nerves as they enter the pathologic target muscle. Additionally, the therapeutic use of Sunderland third-degree nerve injury in the treatment of CD is a useful adjunct to muscles that are nonexpendable as it allows for only partial denervation as opposed to complete denervation with traditional methods.

## Introduction

Cervical dystonia (CD) is the most common form of focal dystonia. This neuromuscular disorder is characterized by sustained contractions of cervical muscles, leading to abnormal head movements and positions like rotation (torticollis) or tilting either laterally (laterocollis), anteriorly (anterocollis), or posteriorly (retrocollis) [1-3]. The involuntary, spasmodic muscle contractions can cause disabling pain impairing daily activities and quality of life.

The first-line treatment is chemodenervation of the spasmodic muscles with botulinum toxin type A [4,5]. In 2017, a Cochrane review of several randomized control trials demonstrated a 19% improvement in symptoms following botulinum toxin type A injection in the treatment of CD [6]. If resistance develops to botulinum toxin type A, type B can be used and has shown treatment efficacy [7]. Unfortunately, symptom relief with chemodenervation is usually incomplete, temporary, and requires repeat injections every three to four months. This treatment also may lead to neck weakness and dysphagia.

Traditional surgical treatment options for CD include deep brain stimulation (DBS), selective peripheral denervation, and myotomy. DBS of the globus pallidus is considered a second-line for patients with CD unresponsive to chemodenervation and randomized trials have shown significant reduction in dystonic symptoms [8]. Patients with tonic dystonia may be good candidates for selective peripheral nerve denervation where nerves to the pathological muscles are transected disrupting axonal transmission and impairing the ability of nerves to regenerate.

Unfortunately, in patients with diffuse disease involving anterior, lateral, and posterior neck muscles, peripheral denervation cannot be performed, as it would cause diffuse neck weakness. Additionally, muscles like the trapezius serve critical functions beyond head movement, so their complete denervation would leave patients without the ability to fully abduct their shoulder or reach behind their heads. Therefore, not all muscles are expendable for peripheral denervation and require other options.

Sunderland classified nerve injuries into five degrees based on the damaged structural component of the nerve [9]. Fifth-degree injuries, comparable to complete transection like those created with selective peripheral denervation, result in complete disruption of all structures, and without nerve repair, regeneration will not occur. Third-degree injuries result in disruption of the axons and endoneurium; however, the perineurium and epineurium remain intact. Without the endoneurium, intrafascicular scarring and fibrosis occur, hindering full axonal regeneration thus resulting in partial recovery of the distal muscle [10].

Both selective peripheral nerve denervation and myotomy are traditionally performed through a posterior approach. Here, we describe a case of selective peripheral nerve denervation using a periauricular approach to access muscles of the posterior and anterior neck. This approach allows for resuspension of ptotic facial tissues from long-standing CD. Additionally, less spasmodic and nonexpendable muscles were treated with an alternative method to complete selective denervation, namely, iatrogenic Sunderland third-degree nerve injury for incomplete denervation.

## Case presentation

Patient history and symptoms

**Figure 1:**
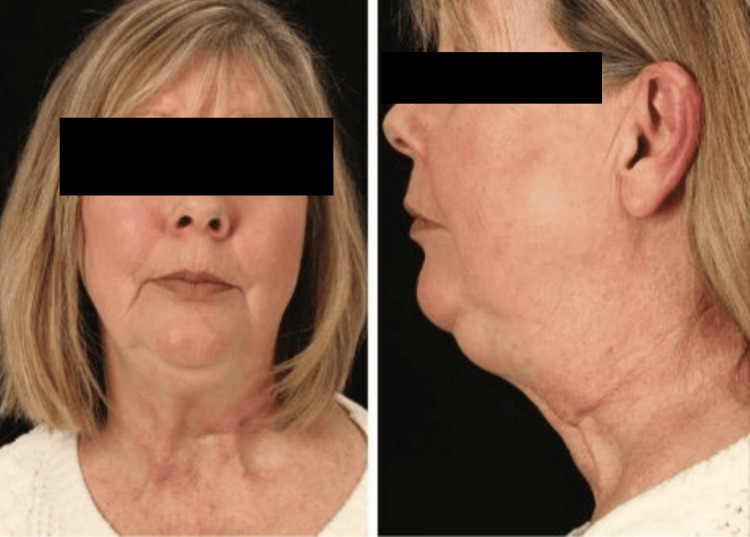
Preoperative frontal and lateral views Preoperative frontal (left) and lateral (right) views of a 58-year-old female with an eight-year history of cervical dystonia with retrocollis and left laterocollis. Platysmal bands from a spasmodic platysma are evident as well as a slight lateral head tilt.

A 64-year-old female presented with an eight-year history of progressive tonic CD, manifesting as painful retrocollis and left laterocollis. She described that her symptoms began as discomfort in the neck, which was followed by posterior neck spasms. Her most distressing symptom was the sensation of her head being forcefully pulled backward due to retrocollis. To bring her neck forward to a more comfortable position, she used her fingers to hook her jaw and pull her head forward, while she supported the back of her head with her other hand. The patient noted her left side was more symptomatic than the right. As her disease progressed, she developed spasms of the bands in her anterior neck from her platysma, significantly affecting her quality of life (Figure 1).

Despite receiving repeat injections of 500 units of botulinum toxin every three months for six years, she only experienced partial and temporary relief. The specific targets for chemodenervation included the semispinalis capitis (25 units/side), splenius capitis (right 25 units, left 50 units), levator scapulae (50 units/side), sternocleidomastoid (SCM) (50 units/side), trapezius (right 50 units, left 75 units), and cervical paraspinals (25 units/side). Chemodenervation was approved by insurance every three months, but relief only lasted six weeks from injection. Therefore, she was seeking a permanent solution.

**Figure 2:**
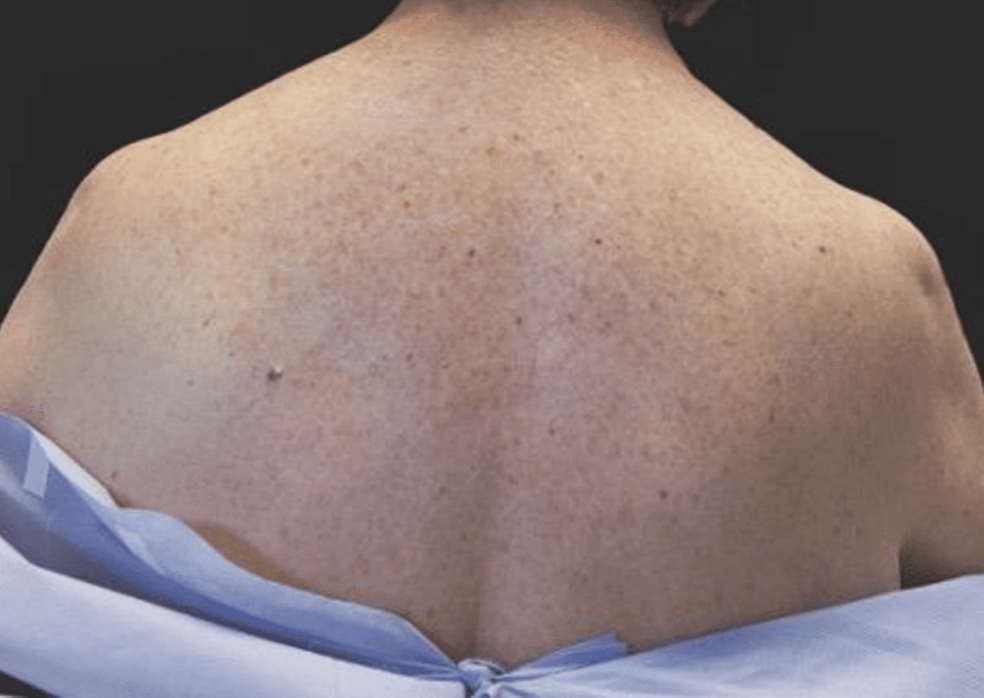
Preoperative posterior view Posterior view of the described patient. Her left shoulder was raised compared to the right with a bulkier trapezius on the left.

Upon examination, the patient's posture was notable--she sat at rest with her hand supporting the back of her head to keep her neck in an upright position. Although her neck and head position were midline at rest, neck extension revealed a leftward tilt and rotation. She had frequent spasms of the platysma bilaterally that worsened with neck extension and speech. Her left shoulder was elevated, and the trapezium muscle appeared bulkier on the left (Figure 2).

Neck range of motion was limited in all directions, a restriction demonstrated in the preoperative examination video (Video 1).

**Video 1:** Preoperative exam Video showing the preoperative exam of the patient described. Preoperatively, her platysmal spasms and decreased neck range of motion are demonstrated.

The patient’s presentation was consistent with CD with retrocollis and left laterocollis. Her spasmodic platysma appeared to be compensatory to counteract her retrocollis, as it worsened with attempted neck extension. To address both the anterior and posterior neck, we opted for a comprehensive surgical approach that included a periauricular incision to perform selective peripheral denervation, selective nerve injury, and myotomies. Selective peripheral denervation was performed for the cervical branch of the facial nerve (to the platysma) and branches of the cervical plexus (semispinalis capitis, longissimus capitis, and levator scapulae). Because the trapezius muscle is necessary for shoulder function and SCM is needed to counteract her retrocollis, we felt an attempt at partial denervation by inducing a Sunderland third-degree nerve injury to result in decreased muscle strength following eventual nerve regeneration should be performed in conjunction with a resuspension of the descended facial tissue, from years of platysma muscle spasms.

Surgical technique

The patient was placed under general anesthesia and positioned supine with the head and neck prepped into the sterile field. Bilateral periauricular incisions were designed anterior to the temporal hairline, down the ear-cheek junction, behind the tragus, under the ear lobule, superiorly along the retroauricular sulcus, and then posteriorly and inferiorly along the occipital hairline down the neck. A submental incision was designed posterior to the submental crease. Epinephrine saline tumescent solution was injected in the subcutaneous dissection plane along marked incisions, cheeks, neck, and mastoid skin to assist with perioperative hemostasis. The submental and left preauricular incisions were made first and dissection was carried down to the superficial musculoaponeurotic system (SMAS) in the face and the platysma in the neck.

Supraplatysmal skin flaps are elevated via the submental incision and carried down to the cricoid cartilage and as far laterally as possible. The left preauricular skin flaps were then elevated anteriorly in the supra-SMAS plane until dissection reached the anterior sideburn. The posterior and inferior mastoid dissection is followed inferiorly until the plane is connected to the one made from the submental incision. Elevation in the supraplatysmal plane was made using Kahn scissors and protected bipolar electrocautery-controlled bleeding. A medially based SMAS flap was designed beginning immediately anterior to the ear and extending medially over the temporal process of the zygoma. The SMAS was incised down to the correct plane, which was identified posteriorly by the white and shiny enveloping layer over the superficial parotid. The SMAS flap was continuous with the platysma, and elevation of the SMAS/platysma flap allowed exposure of lower branches of the facial nerve and resuspension of the ptotic face and platysma at the end of the case. At the inferior aspect of the flap in the sub-SMAS plane, the marginal mandibular and cervical branches of the facial nerve were identified. A nerve stimulator (Checkpoint Surgical, Cleveland, OH) confirmed the identity of both nerves prior to the transection of the cervical branch supplying the platysma.

Following the transection of the cervical branch, stimulation of the proximal nerve branch will elicit no movement of the ipsilateral platysma. The distal end, however, will still cause the firing of the platysma muscles for 72 hours if stimulated.

Dissection then proceeded to the posterior border of the SCM muscle to access the spasmodic posterior triangle muscles, carefully avoiding damaging the great auricular nerve in this location. The semispinalis capitis, levator scapulae, and longissimus capitis were identified with the nerve branches of the cervical plexus as they entered the deep surface of these muscles. Nerve branches to these three muscles were stimulated to confirm identity prior to nerve transection. The phrenic nerve was also identified, stimulated, and protected during dissection. Full-thickness transverse myotomies were then performed on the same three muscles using electrocautery.

Next, the spinal accessory nerve and its branches were identified on the anterior border of the trapezius. These branches were confirmed via a nerve stimulator. A Sunderland third-degree nerve injury was induced on each branch of the SAN by crushing nerves for 30 seconds using toothless Gerald forceps [10]. Successful nerve injury was confirmed by proximal nerve stimulation that elicited no motor response. A step-lengthening myotomy of the SCM was then performed.

Dissection and peripheral denervation proceeded similarly on the right side. However, selective nerve injuries and myotomies were not performed to the right SCM or trapezius since these procedures should be tailored to patients’ specific needs. In this case, these muscles on the right were not hypertrophic or symptomatic.

The ptotic facial tissues were then resuspended to their anatomically normal position. A corset platysmaplasty was performed midline with plication using a 3-0 polydioxanone suture in a running locking fashion via the submental incision. Next, the previously mobilized SMAS flaps were retro-positioned behind the ears with the creation of a small pennant SMAS flap to help resuspend the lower face and neck. The SMAS flap was also sutured to its cut preauricular edge. This was secured to mastoid fascia using a 3-0 polydioxanone suture. Redundant skin was excised, and skin closure was performed with interrupted 3-0 Monocryl and running 5-0 fast-absorbing gut over a closed suction drain. The incisions were dressed with Xeroform gauze and bacitracin ointment, followed by 4 x 4 gauze and a headwrap reinforced with facelift tape.

Postoperative course

The patient remained in the hospital for a 23-hour observation period. On postoperative day one, dressings were removed to examine for a hematoma. The drain was also removed, and she was placed into a jaw bra with sterile dressings over the incisions.

**Figure 3:**
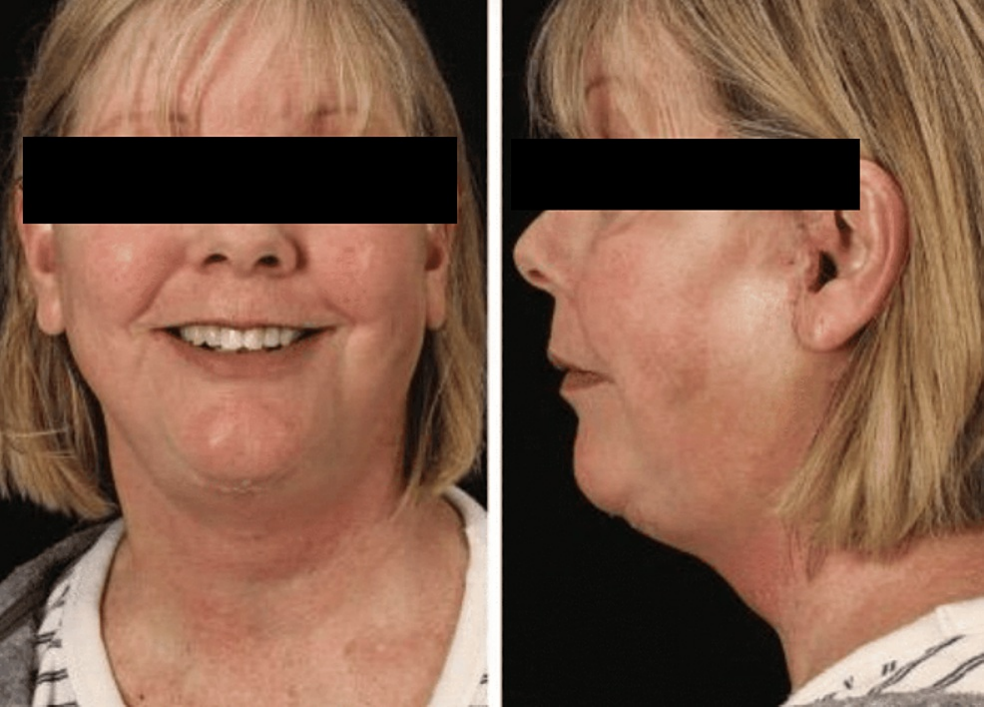
Postoperative frontal and lateral views Postoperative frontal (left) and lateral (right) images of the patient at one-month follow-up. The prominent platysmal bands are now absent with a better-defined cervicomental angle. Her neck is maintained upright without support and she has improved appearance of her mid and lower facial tissue. The preauricular incisions, seen best on the lateral view (right), are healing and are concealed just anterior to the ear and hairline. The postauricular incisions are not visualized.

At one-month post procedure, the patient reported an 80% improvement in pain and symptoms. Her head maintained a better position with an improved range of motion. Her left trapezius was weaker than the right, leaving her shoulders and head in a level position. Spasmodic platysmal bands on the left were absent, even with neck extension. Bands on the right were very diminished (Figure 3).

On the left, she was unable to reach completely behind her head, but she was not bothered by this. No other significant limitations were noted. After this visit, the patient did not return for any subsequent visits.

## Discussion

CD remains challenging to manage, with treatments evolving since the 1800s. Herein, we present another evolution in the technique for selective peripheral denervation via the periauricular approach. This incision allows access to several key areas: the posterior triangle muscles, SCM, trapezius, and anterior platysma muscles, and their respective motor nerve branches. To our knowledge, this is the first description of selective peripheral nerve denervation by this approach. A periauricular incision is traditionally utilized to perform facial reanimation or rhytidectomy and was beneficial in this case to address both the spasmodic posterior neck muscles and platysma. The incision is well concealed and allows for concomitant resuspension of facial tissue descent caused by years of platysmal spasm.

The Sunderland third-degree nerve injury of the spinal accessory nerve was successful in weakening the spasmodic SCM and trapezius muscles through axonotmesis and, as anticipated, reduced functional motor recovery. This incomplete selective peripheral nerve denervation represents a novel modality for the surgical treatment of focal dystonias. The consequences of complete denervation of the trapezius or the SCM include permanent removal of an important shoulder stabilizer (trapezius) and a muscle that can counteract retrocollis (SCM) and be undesirable. The Sunderland third-degree nerve injury allows partial regeneration over the course of six months leading to weaker muscle function.

This description represents a novel approach to the treatment of CD and is particularly useful in patients with both posterior and anterior symptoms. This approach allows for easy identification of nerves as they enter the pathologic target muscle. Additionally, the therapeutic use of Sunderland third-degree nerve injury in the treatment of CD is a useful adjunct to muscles that are nonexpendable as it allows for only partial denervation as opposed to complete denervation with traditional methods.

## Conclusions

We present an anterior approach to CD treatment that leverages a periauricular incision for selective peripheral nerve denervation, along with the strategic application of Sunderland third-degree nerve injury. This technique represents another surgical option for CD, particularly beneficial for patients with diffuse muscle involvement or involvement of muscles necessary for shoulder mobility and head stability. This technique allows for the preservation of non-pathologic muscle function with selective denervation of the involved pathologic muscles by facilitating more precise nerve identification and targeted treatment of spasmodic. The therapeutic use of Sunderland third-degree nerve injury as an adjunct to this approach allows partial denervation of necessary functional muscles, enabling the reduction of spasmodic activity without the drawbacks of complete loss of function. The promising results demonstrated in our case study, including significant symptom improvement and preserved muscle function, underscore the potential of this approach to improve the quality of life of patients with CD.
